# Survival outcomes in patients with stages I–III gastric adenocarcinoma treated with surgery alone versus surgery plus adjuvant chemotherapy: A systematic review

**DOI:** 10.14440/jbm.2025.0135

**Published:** 2025-04-18

**Authors:** Naveena Kaur Rikhraj

**Affiliations:** 1 Department of Medicine, Lancashire Teaching Hospitals NHS Foundation Trust, Preston, Lancashire PR2 9HT, United Kingdom; 2 Department of Medical Sciences, Faculty of Biology, Medicine, and Health, University of Manchester, Manchester M13 9PL, United Kingdom; 3 Department of Medicine, School of Medicine, University of St Andrews, Fife, Scotland KY16 9TF, United Kingdom

**Keywords:** Gastric adenocarcinoma, Adjuvant chemotherapy, Gastrectomy, Disease-free survival, Overall survival, Recurrence-free survival

## Abstract

**Background:**

Gastric adenocarcinoma, the malignant proliferation of glandular cells in stomach epithelium, is a type of gastric cancer with a statistical disease burden of the fifth most common cancer globally and the 17^th^ most common malignancy in the United Kingdom. Prognosis varies with stage (I–IV), with stages I–III showing promising 5-year survival rates up to 71.8%, warranting timely diagnosis and treatment. Surgery is the gold-standard treatment; however, due to complex tumor pathophysiology, there is growing interest in the use of multimodal therapies. Specifically, the combination of surgery and adjuvant chemotherapy has become a key focus of the treatment for stages I–III gastric adenocarcinoma.

**Objective:**

The study reviewed patients with stages I–III (non-advanced) gastric adenocarcinoma to assess whether adjuvant chemotherapy combined with surgery provides better disease-free/disease-specific/cause-specific survival, overall survival, and reduced recurrence rates/improved recurrence-free survival, compared to surgery alone. Analyzed were 17 English-language, full-text, levels I–III peer-reviewed studies from MEDLINE and Embase from the past 10 years were analyzed. No age/sex/ethnicity/country restrictions were applied, and the dimension of interest was limited to stages I–III gastric adenocarcinoma patients who underwent tumor resection through surgery and received chemotherapy as the only adjuvant therapy. Seven (41.2%) studies have more than one statistically significant outcome measure supporting the benefit of adjuvant chemotherapy in combination with surgery over surgery alone. Ten (58.8%) studies showed no statistically significant benefit of adjuvant chemotherapy.

**Conclusion:**

The findings contrasted with previous large-scale meta-analyses, which were limited by sample size and biases in individual studies reviewed. Continued research, incorporating advances in surgical techniques and new chemotherapeutic combinations, is necessary to ascertain best-tailored treatments for gastric adenocarcinoma.

## 1. Introduction

### 1.1. Epidemiology

Gastric adenocarcinoma, localized to the stomach, is the fifth most common cancer globally, accounting for 7.7% of deaths yearly.^1^ The highest incidences worldwide are present in Eastern Asia, followed by Central and Eastern Europe, where these populations show strong dietary association with intake of salted, pickled, and smoked foods, which can form carcinogenic N-nitroso-compounds when reacting with nitrite-reducing bacteria in the stomach.^2^ Other notable risk factors include low vitamin A or C intake, smoking, alcohol consumption, *Helicobacter pylori* infection, autoimmune gastritis, and genetic susceptibility. In the United Kingdom, gastric cancer is the 17^th^ most common cancer, representing 3% of all cancer-caused deaths, with an increasing incidence in a left-skewed distribution annually at a male-to-female ratio of 1.71:1.^3^ Peak incidence in terms of age is 75 – 79 years for males and 80 – 84 years for females.

### 1.2. Etiopathogenesis

Approximately 95% of stomach neoplasms are adenocarcinomas that develop due to the malignant proliferation of glandular epithelium of endodermal or ectodermal origin. Anatomically, the most common sites for gastric adenocarcinoma are the lower third of the stomach (antrum, pylorus, and angle), accounting for 60.6 – 79.4% of cases. The middle third (cardia and fundus) is affected in 14.5 – 32.3% of cases, while the upper third involvement accounts for 4.3 – 7.1%.^4^ Consistent with this, Kang *et al*.^5^ reported common regions for tumor growth include the lower part of the stomach (89.6%) and areas along the lesser curvature (43.6%), as they are more prone to damage by reflux of duodenal contents in chronic conditions, such as ulcer. Hematogenous spread is common, particularly to the liver, lungs, adrenals, bone, and the central nervous system. Roughly 74 – 88% of cases have lymphatic spread, with 14% of early-stage cases demonstrating lymph node involvement.^6^

### 1.3. Diagnostic approach

Clinically, early-stage, localized gastric adenocarcinomas are often asymptomatic. Patients present with the most common clinical signs and symptoms of dyspepsia, weight loss, and ulcer-like epigastric pain. The pain is mild to moderate, gnawing in nature, radiates to the back (if the stomach wall is penetrated posteriorly), and can be exacerbated or relieved by food.^7^ Patients with tumors in the cardiac region, near the gastroesophageal junction, can present with dysphagia. Anemic symptoms, such as iron deficiency and hematemesis, can occur if the tumor has caused a hemorrhage along the blood supply of the stomach.^7^ Notably, upon examination, the later stages show signs of metastasis, including a palpable mass along lymph node landmarks (i.e., left supraclavicular node, left axial node, and ovaries) and skin changes due to paraneoplastic lesions.^7^

The first-line choice of investigation for diagnosis is esophagogastroduodenoscopy. The Japanese Gastric Cancer Association guidelines classify lesions as type 0–III based on endoscopic appearance. Early lesions can present as either protruding/polypoid (Type 0–I), elevated (Type 0–IIa), flat (Type 0–IIb), depressed (Type 0–IIc), or excavated/ulcerated (Type 0–III),^8^,^9^ The standard protocol is to sample tumor regions (usually base and edge if elevated) or complete excision if the lesion is ≤3 cm, localized to the submucosa, and there is no presence of ulceration visually. The sample is then histologically identified as intestinal/diffuse/mixed-type per Lauren classification.^10^,^11^ Intestinal-type gastric adenocarcinoma is strongly linked to longstanding chronic gastritis secondary to *H. pylori* infection. This progression follows the Correa cascade, which describes the transformation from atrophic gastritis to intestinal metaplasia, dysplasia, and finally, gastric adenocarcinoma. Thus, in patients presenting with a longstanding history of gastritis, *H. pylori* testing (stool antigen testing, C-13 urea breath test, rapid urease test for *Campylobacter*-like organisms, and *H. pylori* antigen test) can aid in diagnosis.^12^

Serum biomarkers identified through biopsy, such as human epidermal growth factor receptor 2, programmed cell death protein 1/programmed death-ligand 1 (PD-1/PD-L1), microsatellite instability, *NTRK* mutation, and somatic mutations, defined as tumor mutational burden, have predictive implications and can further dictate therapeutic measures. For example, tumors with *HER2* overexpression can benefit from adjuvant immunotherapy, while PD-1/PD-L1 expression can indicate suitability for targeted monoclonal antibody therapy. Similarly, tumors demonstrating microsatellite instability can benefit from neoadjuvant chemotherapy/immunotherapy alongside surgery and adjuvant chemotherapy.^13^

Further imaging for gastric adenocarcinoma diagnosis includes barium meal X-ray (limited current use) and computed tomography (CT) to identify tumor locations, margins, and lymph node spread, along with positron emission tomography, which may have a more significant role in monitoring post-treatment recurrence through uptake of 2-fluoro-2-deoxy-D-glucose trace.^14^

### 1.4. Staging and prognosis

The tumor, node, metastasis (TNM) staging by the American Joint Committee on Cancer remains the standard for staging gastric adenocarcinoma through CT.^15^ Clinically determined staging can be referred to as cTNM. After specimens have been excised and sent for laboratory investigation, the stage is referred to as pTNM, where p stands for pathological, and is often used for post-surgically removed tumors that require adjuvant chemotherapy. The 5-year general survival prognosis of stages I–III gastric adenocarcinomas is up to 71.8%. Beyond stage IIIc, this statistic drops to 5.9% for stage IV, warranting that early diagnosis and effective treatment of non-metastatic disease can significantly improve prognosis.^15^,^16^

### 1.5. Therapeutic approach

**Figure 1:**
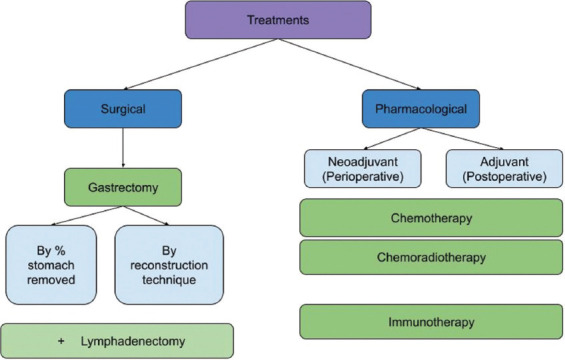
Categorization of treatments for gastric adenocarcinoma

Management of gastric adenocarcinoma can be categorized into surgical and pharmacological management (Figure 1). The gold-standard therapy for gastric adenocarcinoma is resection (R0–3) through a surgical technique of gastrectomy accompanied by reconstruction and/or prophylactic lymphadenopathy (D1–D4).

During gastrectomy (open, laparoscopic, or robotic), surgeons often perform prophylactic lymphadenectomy due to the challenge of accurately predicting lymph nodes that contain malignant cells. The 18 lymph nodes of the stomach are classified into three tiers: Tier 1 (within 3 cm of the primary tumor), Tier 2 (along the main arterial branches), and Tier 3 (non-visible nodes). Lymphadenectomy can be categorized as D1, D2, D3, or D4 in terms of the number of tiers removed: D1 involves the removal of nodes in Tier 1, D2 includes Tier 1 and 2 nodes, D3 removes Tiers 1, 2, and 3, and D4 excises all the above plus paraaortic nodes.^17^ Based on the nodal status of stages I–III gastric adenocarcinoma and as reflected in literature, D1/D2 lymphadenectomy is most commonly performed. According to the present JCGA guidelines,^18^ D2 (+) lymphadenectomy – removing posterior and paraaortic nodes – should be considered for bulky N2 tumors to improve locoregional control.

The preferred procedure nowadays is R0 resection, typically involving distal total gastrectomy, which can extend from gastroesophageal junction to pyloric or antral tumors.^14^ Reconstruction techniques following this procedure include Billroth I (gastroduodenostomy), Billroth II (gastrojejunostomy), and Roux-en-Y anastomosis (gastrojejunostomy to the second jejunal loop with jejunal-jejunal anastomosis of the first and second loops). The reconstruction technique choice depends on the tumor’s location and margin extent. He and Zhou^19^ reported that Roux-en-Y reconstruction, indicated for more distal tumors, improves gastritis and reflux esophagitis symptoms post-operatively due to the diversion of bilo-pancreatic fluids from the gastric outlet. Wang *et al*.^20^ reported 40 – 60% relapse rates across all stages of gastric adenocarcinoma post-initial surgery. Thus, adjuvant chemotherapy after primary surgery has been used in key clinical trials (Table 1) to target cellular mechanisms and optimize therapeutic outcomes.

**Table 1:** Adjuvant chemotherapeutic regimens used in the treatment of stages I–III gastric adenocarcinoma

Regimen	Chemotherapeutic agents
XELOX or CAPOX	Capecitabine+oxaliplatin
ECX or ECF	Epirubicin+cisplatin+capecitabine Epirubicin+cisplatin+5-fluorouracil
UFT	Tegafur+uracil
S-1 or TS-1	Tegafur+gimeracil+oteracil
SOX	S-1+oxaliplatin
DOS	Docetaxel+S-1+oxaliplatin
Fluorouracil derivative+ platinum-coordinate complex	-
5’- DFUR	Doxifluridine
FOLFOX	Oxaliplatin+leucovorin+5-fluorouracil
FLOT	5-fluorouracil+ feucovorin+oxaliplatin+docetaxel
FAM	5-fluorouracil+doxorubicin+mitomycin-c
FOLFIRI	CPT-11 (Irinotecan) + leucovorin+5 -fluorouracil+docetaxel+ cisplatin+dexamethasone

Central to all management options is the involvement of a multidisciplinary team (MDT), which involves radiologists, oncologists, surgeons (gastrointestinal, hepatobiliary, or thoracic, dependent on the extent of invasion), pathologists, anesthesiologists, nutritionists/dieticians, and specialist nurses. The focus is on generating a personalized treatment plan, managing post-operative symptoms, and follow-up staging/remission monitoring. Studies have shown that MDT discussions lead to an approximately 9.5% improvement in the 3-year overall survival (OS) rate (*p*=0.005), which can be attributed to a more thorough follow-up and holistic treatment approach.^21^ This highlights the critical role of the diagnostic process in facilitating thorough and timely management.

## 2. Aims

This study aimed to evaluate two commonly used present treatment modalities by comparing surgery alone with surgery combined with adjuvant chemotherapy in the treatment of stages I to III gastric adenocarcinoma. Specifically, it investigated whether surgery alone results in (i) greater disease-free survival (DFS)/disease-specific survival/cause-specific survival, (ii) OS, and (iii) reduced recurrence rates or improved recurrence-free survival (up to 5 years), compared to surgery plus adjuvant chemotherapy. The study’s inclusion criteria aimed to provide an updated literature review, particularly evaluating whether these established treatment modalities have a combinatory effect on accelerated patient outcomes.

## 3. Methods

### 3.1. Search strategy

**Figure 2:**
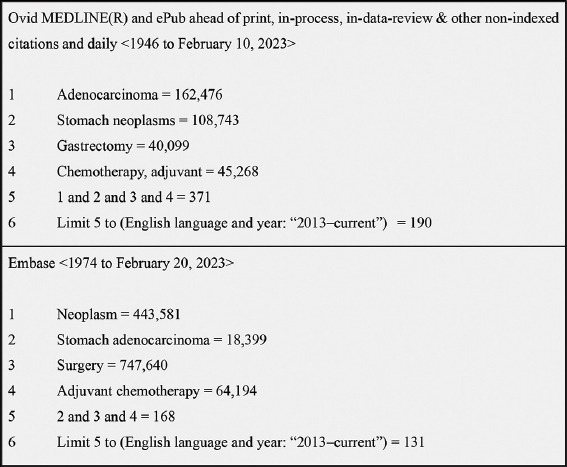
The search strategy used to obtain initial articles on MEDLINE and Embase databases

Two databases, MEDLINE and Embase, were used to conduct the initial literature search. The number of results returned by the keywords differed between the two databases (Figure 2). The keywords “stomach adenocarcinoma” or “adenocarcinoma” AND “stomach neoplasms” were used. With these keywords, “gastrectomy” OR “resection” OR “surgery” AND “adjuvant chemotherapy” were used to obtain approximately <400 papers from each database. Further limits, such as English and articles published in the past 10 years (2013 onward), yielded under 200 papers per database for further screening. With this, a more streamlined question was formulated in Table 2 to narrow down papers using the population, intervention, comparison, and outcome criteria.

**Table 2:** Formulation of questions for investigation

Criteria	Description
Population	Patients diagnosed with stages I to III gastric adenocarcinoma
Intervention	Surgery (gastrectomy with or without lymphadenectomy)
Comparison	Surgery combined with adjuvant chemotherapy
Outcomes	• Disease-free survival (1 – 5 years post-treatment)
	• Recurrence-free survival (1 – 5 years post-treatment)
	• Disease-specific/cause-specific survival (1 – 5 years post-treatment)
	• Overall survival (1 – 5 years post-treatment)
	• Recurrence rates (%)

### 3.2. Selection criteria and preferred reporting items for systematic reviews and meta-analyses flow diagram

**Figure 3:**
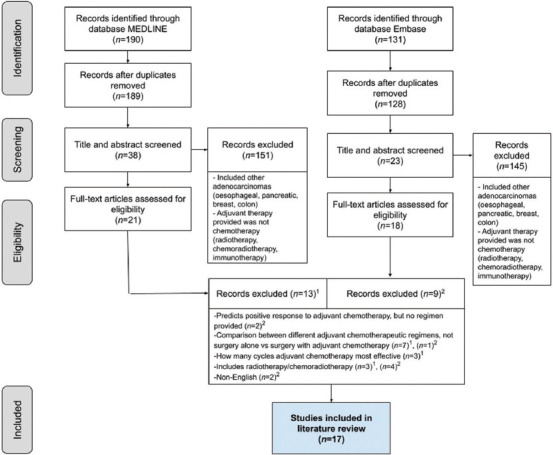
Preferred reporting items for systematic reviews and meta-analyses flow diagram. The flow diagram was used to identify the 17 studies included in the literature review.

As a secondary process, several inclusion and exclusion criteria were employed (Table 3) to filter articles, resulting in a total of 17 articles (Figure 3), which consisted of three experimental/intervention studies comprising randomized-control trials (level I evidence) and 14 observational studies (10 cohort studies [level II evidence] and four case-control [level III evidence]). Study quality and bias were assessed using the Critical Appraisal Skills Programme appraisal tool, and bias plots were generated visually before inclusion.

**Table 3:** Inclusion and exclusion criteria according to article specifics and dimension of interest

Criteria	Inclusion criteria	Exclusion criteria
Article specifics	• English-language	• Non-English articles
	• Full-text	• Systemic reviews/meta-analyses
	• In peer-reviewed journals	• Non-human participants
	• Published in the past 10 years (2013 to present)	
	• Articles with levels I to III on the evidence hierarchy, with primary research (qualitative, quantitative, or mixed data), for example, clinical trials	
	• Any participant group, regardless of age, sex, or ethnicity	
	• All population groups and countries (regional/international)	
Dimension of interest	• Patients with a diagnosis of stages I–III gastric adenocarcinoma	• Stage IV gastric adenocarcinoma
	• Patients who had undergone resection of the tumor through surgery	• Articles where patients present with any adenocarcinoma aside from gastric
	• Patients with only gastric adenocarcinoma	• Endoscopic/submucosal resection of tumors
	• Patients receiving adjuvant chemotherapy (after surgery)	• Chemotherapy provided to patients non-adjunctly, that is, neoadjuvant/ preoperatively
	• Patients receiving chemotherapy as the only form of adjuvant therapy	• Patients receiving more than one form of adjuvant therapy, that is, radiotherapy, chemoradiotherapy, immunotherapy

### 3.3. Outcome measures and statistical analysis

All 17 papers included for review had at least one of the pre-defined outcome measures: (i) greater DFS/disease-specific survival/cause-specific survival, (ii) OS, or (iii) decreased recurrence rates or improved recurrence-free survival (up to 5 years), with 14/17 (82.4%) comparing two outcome measures. Five papers (two randomized controlled trials, two cohorts, and one case-control) defined primary and secondary endpoints.

Descriptive statistics (mean, median, ranges, and interquartile ranges) were used for raw data analysis of confounding variables, prognostic factors, risk factors, and patient demographics. All papers analyzed primary outcomes (survival analysis) using Kaplan-Meier survival estimations or Cox proportional hazards regressions, with *p*-values and hazard ratios selected to assess the significance of results across exposure and control groups.

## 4. Results

**Figure 4:**
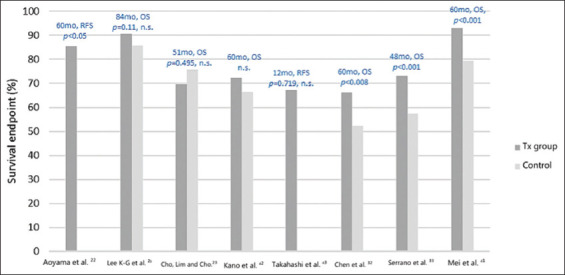
Survival endpoints according to percentage in treatment and control groups. The treatment (tx) group is defined as surgery plus at least one course of adjuvant chemotherapy regimen. Abbreviations: mo: Months; n.s.: Not significant; OS: Overall survival; RFS: Relapse-free survival.

**Figure 5:**
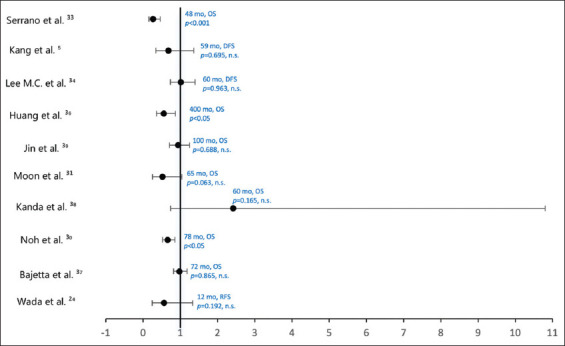
Forest plot showing survival according to hazard ratios (95% confidence interval) Abbreviations: DFS: Disease-free survival; mo: Months; n.s.: Not significant; OS: Overall survival; RFS: Relapse-free survival.

Seventeen studies^22^-^24^, ^29^-^34^, ^36^-^43^ identified and filtered through a literature search were compared using two tables: Table S1, summarizing tumor characteristics and interventions received in studies included for review, and Table S2, analyzing outcome measures and factors favoring treatment. Figure 4 summarizes the primary survival endpoints of relapse-free survival (RFS) and OS according to percentage in treatment and control groups. The treatment group is defined as surgery plus at least one course of adjuvant chemotherapy regimen. Figure 5 is a forest plot showing survival (OS, RFS, and DFS) according to hazard ratios (95% confidence interval).

According to the OS as a primary endpoint, over a follow-up period of 48 – 400 months, seven (41.2%) studies reviewed demonstrated, with statistical significance, that surgery in combination with adjuvant chemotherapy was more effective than surgery alone for the treatment of stages I–III gastric adenocarcinoma. Three (17.7%) of these studies showed significant improvement in OS in treatment groups that received at least one course of adjuvant chemotherapy compared to surgery alone. The study by Aoyama *et al*.^22^ was disregarded due to a lack of data compared to a control group. When hazard ratios were evaluated, three studies (17.7%) reported hazard ratio <1, with statistical significance, meaning the treatment group studied had a decreased probability of an adverse event, thus improved survival, compared to the surgery alone control groups. Based on individual outcome measures (Table S2), 16 (94.1%) of the papers reviewed indicated that groups provided with adjuvant chemotherapy showed some improvement compared to groups with surgery alone. However, with no statistical significance, these values are disregarded in terms of generalizability and application to the sample populations in question.

## 5. Discussion

It can be summarized that the majority of papers do not show that adjuvant chemotherapy combined with surgery offers (i) greater DFS/disease-specific survival/cause-specific survival, (ii) OS, or (iii) decreased recurrence rates or improved RFS (up to 5 years), compared to surgery alone for the treatment of stages I to III gastric adenocarcinoma. Findings from individual analysis in this review revealed that within these sub-stages, small T1N2M0/T1N3M0 tumors had poor overall prognosis after both treatments.^23^ Findings from Wada *et al*.^24^ also showed that tumor diameter was an independent predictor for recurrence after adjuvant S-1 chemotherapy in patients who had undergone surgical resection. This study was not contemplated due to insufficient access to raw data, including propensity score and substage analysis of treatment effect across stages I–III gastric adenocarcinoma.

When larger-scale systematic reviews, such as Diaz-Nieto *et al*.^25^ comprising 34 randomized controlled trials with 7,824 participants, were compared, there was a statistically significant benefit of adjuvant chemotherapy on OS. Fifteen trials (comprising 4,133 participants) showed statistically significant DFS. While these trials investigated all stages of gastric cancer, Peters^26^ noted no significant difference in the stage or chemotherapy regimen provided to patients. Older meta-analyses such as Mari *et al*.^27^ reported that adjuvant chemotherapy reduced the risk of death by 18% but showed discrepancies in the significance of specific chemotherapeutic regimens used (5-fluorouracil and anthracyclines, for example, epirubicin did not show an improvement). The GASTRIC group meta-analysis from 2010^28^ with 6,390 patients showed a statistically significant benefit of fluorouracil adjuvant chemotherapy on OS and reduced risk of death.

Thus, the difference between the findings of this study and the published literature can be attributed to several limitations of this review. With only 17 papers reviewed from two databases, a significant limitation was a low sample size. Given that most papers were location-based in Eastern Asian countries, several papers that met the selection criteria were excluded as they were not in English. Hence, the lack of translation and language bias could have resulted in a loss of pertinent results related to the research question. In line with strong epidemiological and dietary risk-factor associations, the incidence of gastric cancer in Eastern Asia, followed by Central Asia and Eastern Europe – the most recent literature included in this review represents a strongly Eastern approach to treatment for gastric adenocarcinoma. Thus, landmark papers representing Western approaches may have differing implications.

Further strict inclusion and exclusion criteria, particularly in the publication year, excluded earlier trials that showed substantial benefit of adjuvant chemotherapy after surgery to treat gastric adenocarcinoma. Although all papers analyzed in this review meticulously referenced these papers, it would have been interesting to examine differences in methodology, results, and significance of findings between recent literature and these larger trials. Searching more databases and extending the search years is plausible as interventions (particularly chemotherapeutic drugs used) have not changed significantly. Conversely, advances in surgical techniques (laparoscopy and robotic surgery) may improve patient outcomes, lessening the need for adjuvant chemotherapy. A comparison evaluating the role of serum biomarker identification in more targeted novel therapies, such as neoadjuvant, would determine whether it reduces tumor burden and improves post-surgical outcomes, thereby impacting the need for adjuvant therapies. Within this sphere, comparing outcomes in patient groups receiving neoadjuvant or adjuvant chemotherapy in addition to surgery could highlight differing effects of treatment modalities on survival statistics and longevity and help classify the benefiting patient groups. Overall, the sample size can be increased for review, but it should include new and up-to-date published results.

Restricting the analysis to stages I–III gastric adenocarcinoma limited the assessment of adjuvant chemotherapy effects across all stages. While results tables were designed to highlight key survival measures for comparison, reporting bias was inherent due to the variability of outcomes present in the studies. Therefore, the values selected may not fully represent all factors investigated in each of the individual papers. This is coupled with a lack of in-depth statistical analysis of all outcome measures present in the papers. Prognostic and independent risk factors were measured in multiple papers as factors that can influence recurrence rates or occurrence of an event such as death, thereby affecting the effectiveness of adjuvant chemotherapy. However, these were not considered in this review. While a column for factors favoring treatment was added, only univariate statistical values with significance or significantly low hazard ratio values (<1) were selected for consistency. Thus, to draw more plausible conclusions between adjuvant chemotherapy plus surgery on treatment outcomes and surgery alone, bivariate or multivariate analysis should be conducted, and the data can be stratified into several subcategories to increase the accuracy of reported results.

In terms of specific outcome measures, OS was defined as the time from randomization to date of death from any cause, as the primary endpoint in some of the studies,^29^-^33^ making it difficult to distinguish between the OS being cause-related (as a direct result of cancer) or due to secondary non-malignant processes or poor treatment response (i.e., to chemotherapy). Secondary endpoints in Moon *et al*.^31^ were defined by DFS, RFS, site of relapse, and relapse rate. Conversely, Noh *et al*.^30^ defined their primary endpoint as DFS and secondary endpoints as OS and safety. Lee *et al*.^34^ also defined their primary endpoint as DFS, and while Aoyama *et al*.^22^ defined their primary endpoint as DFS, the quantitative values in the paper accounted for RFS instead. According to Oba *et al.*,^35^ there was a very close association between DFS and OS (Spearman correlation coefficient = 0.974, 95% confidence interval = 0.971 – 0.76) (R^2^ almost = 1) as outcome measures in adjuvant trials for gastric cancer, indicating that DFS can similarly represent variability in treatment effects measured by OS. Thus, DFS can reduce trial duration when patients experience adverse cancer-related effects (rather than lifetime duration, which can depend on several confounders), reduce overall trial costs, and minimize data loss due to decreased patient compliance or low follow-up rates. The realization of DFS being an effective survival analysis measure over OS was noted by Aoyama *et al*.^22^ However, this paper was subject to high classification bias as measurement methods were changed toward the end of the study (initially, OS was intended to be reported, followed by DFS, but statistical data were only provided for RFS). Finally, across both OS and DFS, discrepancies were noted in the duration for which the outcome measures were studied. While many papers reported 5-year DFS/RFS and OS rates, most data in graphs and tables accounted for different values (in months), often not extending to 5 years. Huang *et al*.^36^ used a nomogram (computer-generated predictive tool) to predict the 5-year OS based on patient characteristics classified as “high/low-benefit.” While objective, this method implied that patients did not actually undergo the study in exposure and control groups for the intended durations, resulting in potentially low generalizability, high selection bias (participants with demand characteristics), and classification bias.

Critical appraisal of present studies is also necessary to recognize future research directions. Multicenter, multicountry studies, such as the one conducted by Noh *et al.*,^30^ which adjusted for prognostic factors with multivariate analysis, provided a model for further sample study design. Accounting for balanced, cross-matched, and blinded patient exposure and control groups studied in a prospective study could offer a more representative analysis of treatment effects. As survival statistics are key tools for assessing treatment effectiveness, generating streamlined guidelines for the frequency and duration of follow-up will enable a more reliable evaluation of the most effective, up-to-date treatments for stages I–III gastric adenocarcinoma.

## 6. Conclusion

The results from the 17 studies reviewed showed no statistically significant benefit of adjuvant chemotherapy after surgery for the treatment of stages I–III gastric adenocarcinoma, compared to surgery alone. These findings contradict large-scale meta-analyses that have established significant benefits of adjuvant chemotherapy beyond the past 10 years. However, the discrepancy may stem from the selective nature of this study and its specific aims, underscoring the need for more in-depth statistical analysis and expanded selection criteria to include all substages, among other limitations. Given the recent advances in surgical techniques, the application of new chemotherapeutic combinations, and the mounting emphasis on MDT involvement, ongoing research is essential to the determination of the efficacy of multimodal treatments alongside surgery as the cornerstone for gastric adenocarcinoma management.

## Data Availability

Not applicable.

## Supplementary file

**Table S1:** Specifications of each study according to patient demographics, tumor characteristics, and interventions

Study	Study type and location	Patient demographics	Primary tumor characteristics	Surgical intervention *n* (%)	Adjuvant chemotherapy regimen
Bajetta *et al*. 1	RCT, Italy	*n*=1,100 Median age (range), years=62 (23.6 – 77.0) Sex ratio (male: Female)=1.73: 1	TNM stage, *n* (%) • IB=88 (8.1) • II=346 (31.8) • IIIA=297 (27.3) • IIIB=157 (14.4) • III+/unknown=212 (19.27) Tumor location, *n* (%) • Upper third=158 (14.9) • Middle third=248 (26.7) • Lower third=339 (31.9) • Other/unknown=319 (29) Histological classification, *n* (%) • Intestinal-type=303 (33.2) • Diffuse-type=340 (37.2) • Mixed=73 (8.0) Other/unknown=387 (34.91)	Gastrectomy • Total=599 (54.8) • Distal=446 (40.8) • Upper=48 (4.4) • Unknown=7 (0.006) Lymph-adenectomy • D1=272 (25.1) • D2=779 (72.1) • D3=30 (2.8) • Unknown=19 (0.017) • Median lymph nodes removed per patient=27	FOLFIRI Four cycles, each 14 days • Day 1: CPT-11 (180 mg/m^2^, 1 h infusion) • Day 1 – 2: LV (100 mg/m^2^, 2 h infusion) • Day 1 – 14: 5-FU (400 mg/m^2^ bolus, followed by 22 h infusion of 600 mg/m^2^) 3-week break Three cycles, each 21 days • Day 1: Docetaxel (75 mg/m^2^, 1 h infusion) with dexamethasone (before, 12 h and 24 h) • Day 1 – 21: Cisplatin (75 mg/m^2^) ControlNine cycles, each 14 days • Day 1 – 2: LV (100 mg/m^2^, 2 h infusion) • Day 1 – 14: 5-FU (400 mg/m^2^ bolus, followed by 22 h infusion of 600 mg/m^2^)

Noh *et al*. 2	RCT, China, South Korea, Taiwan	*n*=1,035 Age, years=≥18 to≥65 Sex ratio (male: female)=2.4: 1	TNM stage, *n* (%) • IB=1 (<1) • II=514 (49.7) • IIIA=377 (36.4) • IIIB=142 (13.7) • III+/unknown=1 (<1) Tumor location, *n* (%) • Fundus=86 (8.3) • Body=338 (32.7) • Antrum=471 (45.5) • Whole gastric=12 (1.2) • Other=128 (12.4)	Gastrectomy with D2 lymph-adenectomy • 1,035 (100)	Eight cycles, each 3 weeks • Day 1: Oxaliplatin (130 mg/m^2^, intravenous) • Day 1 – 14: Capecitabine (1,000 mg/m^2^, twice daily, oral)

Moon *et al*. 3	RCT, Japan	*n*=229 Median age (range), years=63 (34 – 79) Sex ratio (male: female)=3.23: 1	TNM stage, *n* (%) • IB=103 (45) • II=84 (36.7) • IIIA=42 (18.3)	Gastrectomy, R0 • Total=74 (32.3) • Distal=152 (66.4) • Proximal=48 (3.1) • Other=2 (0.01)	Once daily for 12 months; Either of three fluoropyrimidines: • 5-FU, *n* (%)=31 (13.5) (150 mg ≤50 kg or 200 mg>50 kg, oral) • 5’- DFUR, *n* (%)=42 (18.3) (600 mg ≤50 kg or 800 mg>50 kg, oral) • UFT, *n* (%)=40 (17.5) (300 mg≤1.3 m^2^ BSA or 400 mg >1.3 m^2^ BSA, oral)

Kanda *et al*. 4	Cohort, Japan	*n*=171 Mean age, years=64.8 Sex ratio (male: female)=2.89: 1	TNM stage, *n* (%) • IIA=20 (11.7) • IIB=49 (28.7) • IIIA=28 (16.4) • IIIB=43 (25.2) • IIIC=31 (18.1) Tumor location, *n* (%) • Upper third=45 (26.3) • Middle third=57 (33.3) • Lower third=64 (37.4) • Whole gastric=5 (3) Histological classification, *n* (%) • Well-differentiated=5 (2.9) • Moderately differentiated=54 (31.6) • Poorly-differentiated=103 (60.2) • Signet ring cell=5 (3) • Mucinous=4 (2.3)	Gastrectomy, R0 with D2 lymph-adenectomy • Total=69 (40.4) • Distal=102 (59.7)	4 weeks, followed by a 2-week break, for 12 months: • S-1 (<1.25 m^2^ BSA=80 mg, once daily, oral) (1.25 m^2^ – 1.5 m^2^ BSA=100 mg, once daily, oral) (≥1.5 m^2^ BSA=120 mg, once daily, oral)

Lee *et al*. 5	Cohort, Republic of Korea	*n*=630 Mean age, years=58.1 Sex ratio (male: female)=1.71: 1	TNM stage, *n* (%) Stage IIA: • T3N0M0=387 (61.4) • T2N1M0=161 (25.6) • T1N2M0=82 (13) Tumor location, *n* (%) • Antrum=366 (58.1) • Gastroesophageal Junction=77 (12.2) • Other=187 (29.7) WHO histological classification, *n* (%) • Differentiated=269 (42.7) • Undifferentiated=361 (57.3)	Gastrectomy with non-specified lymph node dissection • 630 (100)	Either of three regimens: • 5-FU single, *n* (%)=65 (33.2) (no doses) • 5-FU combination (with platinum), *n* (%)=127 (64.8) (no doses) • Unknown, *n* (%)=4 (2) (no doses)

Cho *et al*. 6	Cohort, Republic of Korea	*n*=206 Mode age, (range) years=<65 (< 65 to≥65) Sex ratio (male: female)=1.9: 1	TNM stage, *n* (%) • IIA=64 (31.1) • IIIB=58 (28.2) • IIIC=84 (40.8)	Gastrectomy, R0 with≥D2 lymph-adenectomy • 206 (100)	XELOX Eight cycles, each 14 days • Day 1: Oxaliplatin (130 mg/m^2^, intravenous) • Day 1 – 14: Capecitabine (1,000 mg/m^2^, twice daily, oral) Control, TS-1 6-week cycles for 12 months 4 weeks, then a 2-week break: - TS-1 (80, 100, or 120 mg depending on BSA, two separate doses, oral)

Jin *et al*. 7	Cohort, China	*n*=1,601 Mode age, (range) years=≥65 (<65 – ≥65) Sex ratio (male: female)=1.73: 1	TNM stage, *n* (%) Stage IA and Stage IB: • T1N0M0=1,157 (72.1) • T2N0M0=447 (28) Tumor location, *n* (%) • Cardia=392 (24.5) • Fundus=55 (3.4) • Body (lesser curvature)=97 (6.1) • Body (greater curvature)=318 (19.9)	Gastrectomy with D1–D2 lymph-adenectomy • 1,601 (100)	Four–eight cycles, median six. Duration per cycle not specified. Either of four regimens: • S-1 single, *n* (%)=40 (14.1) (no doses) • S-1+oxaliplatin (SOX), *n* (%)=180 (63.6) (no doses) • Capecitabine+oxaliplatin, *n* (%)=30 (10.6) (no doses) • Docetaxel, oxaliplatin+S-1 (DOS), *n* (%)=33 (11.7) (no doses) •Antrum=664 (41.5) • Other=75 (4.7) Histological classification, *n* (%) • Intestinal-type=423 (26.4) • Diffuse-type=366 (22.9) • Mixed=42 (2.6) • Undefined=770 (48.1)

Huang *et al*. 8	Cohort, China	*n*=1,593 Mode age, (range) years=>65 (≤65 – > 65) Sex ratio (male: female)=1.56: 1	TNM stage, *n* (%) Stage IIA: • T1N2M0=158 (9.9) • T3N0M0=1,435 (90.1) Tumor location, *n* (%) • Upper third=788 (49.5) • Middle=241 (15.1) • Lower third=564 (35.4) Histological classification, *n* (%) • Intestinal-type=276 (17.3) • Non-intestinal-type=1,317 (82.7)	Gastrectomy • Total=728 (45.7) • Distal=652 (41) • Upper=129 (8.1) • Antrum=84 (5.3)	(Not specified)

Lee *et al*. 9	Cohort, Republic of Korea	*n*=983 Mode age, (range) years=≥60 (<60 – ≥60) sex ratio (male: female)=2.2: 1	TNM stage, *n* (%) Stage II • T3N0M0=983 (100) Tumor Location, *n* (%) • Upper third=788 (49.5) • Middle=241 (15.1) • Lower third=564 (35.4) Histological classification, *n* (%) • Intestinal-type=346 (35.2) • Diffuse-type=442 (45) • Mixed=96 (9.8)	Gastrectomy, R0 with ≥D2 lymph-adenectomy • Subtotal=642 (65.3) • Total=341 (34.7)	S-1 Eight cycles, 6 weeks each 4 weeks: • S-1 (40, 50, or 60 mg depending on BSA, twice daily, oral) 2-week break Control, CAPOX Eight cycles, 3 weeks each • Day 1: Oxaliplatin (130 mg/m^2^, intravenous) • Day 1 – 14: Capecitabine (1,000 mg/m^2^, twice daily, oral)

Chen *et al*. 10	Cohort, China	*n*=428 Median age (range), years=60 (23 – 79) Sex ratio (male: female)=2.5: 1	TNM stage, *n* (%) • IIA=91 (21.3) • IIB=75 (17.5) • IIIA=139 (32.5) • IIIB=89 (20.8) • IIIC=34 (7.9) Histological classification, *n* (%) • High-differentiation=3 (0.7) • Median-differentiation=84 (19.6) • Low-differentiation=173 (40.4) • Signet ring cell=22 (5.1) • Mucinous=14 (3.3) • Other=132 (30.8)	Gastrectomy with D2 Lymph-adenectomy • 428 (100)	Either of three groups: Group B (20 – 24 weeks), *n* (%)=175 (51.2) • SOX, seven–eight cycles every 3 weeks Day 1: Oxaliplatin (130 mg/m^2^, intravenous over 2 h in 250 mL dextrose 5%) Day 1 – 14: S-1 (40–60 mg depending on BSA, twice daily, oral) • XELOX, seven–eight cycles every 3 weeks Day 1: Oxaliplatin (130mg/m^2^, intravenous over 2 h in 250 mL dextrose 5%) Day 1 – 14: Capecitabine (1,000 mg/m^2^, twice daily, oral) • FOLFOX, 10–12 cycles every 2 weeks Day 1: Oxaliplatin (85 mg/m^2^) and leucovorin calcium (intravenous over 2 h in 250 mL dextrose 5%) 5-FU (400 mg/m^2^, bolus, then 2,400 mg/m^2^, intravenous over 46 h) Group C (12 – 18 weeks), *n* (%)=107 (31.3) • SOX, four–six cycles every 3 weeks (same dosage as Group B) • XELOX, four–six cycles every 3 weeks (same dosage as Group B) • FOLFOX, six–nine cycles every 2 weeks (same dosage as Group B) • Group D (<12 weeks), *n* (%)=60 (17.5) • SOX, ≤3 cycles every 3 weeks (same dosage as Group B and C) • XELOX, ≤3 cycles every 3 weeks (same dosage as Group B & C) • FOLFOX, ≤5 cycles every 2 weeks (same dosage as Group B and C)

Kang *et al*. 11	Cohort, Republic of Korea	*n=*1,083 Mean age, years=58 Sex ratio (male: Female) = 2: 1	TNM stage, *n* (%) Stage II • T3N0M0=1,083 (100) Tumor location, *n* (%) • Upper third=244 (22.5) • Middle=363 (33.5) • Lower third=462 (42.7) • Whole gastric=14 (1.3) Histological classification, *n* (%) • Intestinal-type=409 (37.8) • Diffuse-type=658 (60.8) • Mixed=16 (1.5)	Gastrectomy • Subtotal=676 (62.4) • Total=407 (37.6) Lymphadenectomy • D1+=26 (2.4) • ≥D2=1,057 (97.6) • Mean lymph nodes removed per patient=41.9	Either of five regimens: • UFT, oral (no *n*, no doses) • TS-1, oral (no *n*, no doses) • 5-FU with leucovorin (FL), intravenous (no *n*, no doses) • 5-FU with platinum (FP), intravenous (no *n*, no doses) • Capecitabine with platinum (XELOX) (no *n*, no doses)

Serrano *et al*. 12	Cohort, Peru	*n*=173 Median age, years=62.3 Sex ratio (male: Female)=1.25: 1	TNM stage, *n* (%) • IIA=18 (10.2) • IIB=32 (18.8) • IIIA=65 (36.9) • IIIB=30 (17.6) • IIIC=28 (16.5) Tumor location, *n* (%) • Fundus=3 (1.7) • Body=31 (17.9) • Antrum=116 (67) • Whole gastric=1 (0.7) • Other=22 (12.7) Histological classification, *n* (%) • Intestinal-type=82 (47.4) • Diffuse-type=68 (39.3) • Mixed=23 (13.3)	Gastrectomy with D2 lymph-adenectomy • 173 (100)	≥6 courses, 3 weeks each • Day 1: Oxaliplatin (130 mg/m^2^, intravenous) • Day 1 – 14: Capecitabine (1,000 mg/m^2^, twice daily, oral)

Mei *et al*. 13	Cohort, China	*n*=307 Median age (range), years=63 (29 – 80) Sex ratio (male: Female)=2.4: 1	TNM stage, *n* (%) Stage I • T2N0M0=307 (100) Tumor location, *n* (%) • Upper third=55 (17.9) • Middle=46 (15) Lower third=206 (67.1) Histological classification, *n* (%) • Poor-differentiation=96 (31.3) • Tubular=160 (52.1) • Signet-ring cell=32 (10.4) • Mucinous=19 (6.2)	Gastrectomy, R0 with D2 lymph-adenectomy • Distal=224 (73) • Total=83 (27)	One regimen of: Every 3 weeks for 1 year • S-1 monotherapy, *n* (%)=130 (66.3) (40 mg/m^2^, twice daily, oral) Combined with either of the following dual-drug regimens, *n* (%)=66 (33.7): Six cycles, 3 weeks each • XELOX Day 1: Oxaliplatin (130 mg/m^2^, intravenous) Day 1 – 14: Capecitabine (1,000 mg/m^2^, twice daily, oral) • SOX Day 1: Oxaliplatin (130 mg/m^2^, intravenous) Day 1 – 14: S-1 (40 mg/m^2^, twice daily, oral)

Wada *et al*. 14	Case-control, Japan	*n*=77 Median age (range), years=65.5 (42 – 80) Sex ratio (male: Female)=1.9: 1	TNM stage, *n* (%) • IIA=11 (14.3) • IIB=18 (23.4) • IIIA=14 (18.2) • IIIB=12 (15.6) • IIIC=22 (28.6) Histological classification, *n* (%) • Differentiated=35 (45.5) • Undifferentiated=41 (53.2)	Gastrectomy, R0 with ≥D1–D2 lymph-adenectomy • Partial=38 (49.4) • Total=39 (50.6)	S-1 4 weeks, followed by 2-week intervals, for 1 year • 80 mg/m^2^, once daily, oral

Aoyama *et al*. 15	Case-control, Japan	*n*=52 Median age (range), years=64 (37 – 80) Sex ratio (male: Female)=1.6: 1	TNM stage, *n* (%) Stage II: • T1N2M0=15 (28.8) • T1N3M0=13 (25.1) • T3N0M0=24 (46.1) Tumor location, *n* (%) • Upper third=10 (19.2) • Middle third=27 (51.9) Lower third=15 (28.8) Histological classification, N (%) • Differentiated=25 (48.1) • Undifferentiated=27 (51.9)	Gastrectomy with D1+ – D2 Lymph- adenectomy • 52 (100)	(Not specified)

Kano *et al*. 16	Case-control, Japan	*n*=390 Median age (range), years=64 (27 – 80) Sex ratio (male: Female)=2.3: 1	TNM stage, *n* (%) • IIA=25 (6.4) • IIB=110 (28.2) • IIIA=78 (20) • IIIB=83 (21.3) • IIIC=94 (24.1) Tumor location, *n* (%) • Upper third=109 (27.9) • Middle=159 (40.8) • Lower third=109 (27.9) • Whole gastric=13 (3.3) Histological classification, *n* (%) • Differentiated=135 (34.6) • Undifferentiated=255 (65.4)	Gastrectomy, R0 • Distal=225 (57.7) • Total=165 (42.3)	S-1 4 weeks, followed by 2-week rest, for 1 year (eight courses) • 80 – 120 mg/m^2^, once daily, oral

Takahashi *et al*. 17	Case-control, Japan	*n*=396 Median age (interquartile range), years^a^=63 (56 – 71) Sex ratio (male: Female)^a^=2.2: 1	TNM stage, *n* (%) • IIA=25 (6.3) • IIB=116 (29.3) • IIIA=78 (19.7) • IIIB=83 (20.9) • IIIC=94 (23.7) Histological classification, *n* (%)^‡^ • Differentiated=42 (34.4) • Undifferentiated=80 (65.6)	Gastrectomy (distal/total) with D2 lymph-adenectomy • 396 (100)	S-1 4 weeks, followed by 2-week rest, for 1 year (eight courses) • 80 – 120 mg/m^2^, once daily, oral

Note:

a Refers to data from subgroup analysis of patients who experienced recurrence (n=122) after receiving adjuvant chemotherapy.

Abbreviations: 5-FU: 5-fluorouracil; BSA: Body surface area; CSS: Cause-specific survival; DFS: Disease-free survival; DFUR: 5’- deoxy-5-fluorouridine; h: Hour; HR: Hazard ratio; OS: Overall survival; RCT: Randomized controlled trial; RFS: Recurrence-free survival; UFT: Tegafur/uracil.

**Table S2:** Summary of main results comparing exposure group to reference group with outcome measures

Author	Exposure group, treatment (tx) and reference group, C, n (%)	Controlled confounding variables	Outcome measures	Reference *P-*value for significance	Factors favoring treatment	Adverse effects, *n* (%)	Conclusions
Bajetta *et al*. 1	(Tx) FOLFIRI 562 (51.1) (C) 538 (48.9)	· Age ≤75 years • pT2b/pT3 pN- or pN+, Stage II and III • Gastrectomy with ≥D1 lymphadenectomy • ECOG <2 • No previous malignancy except superficial skin cancer and *in situ* cervical carcinoma • No previous chemotherapy and radiotherapy • No abnormal hepatic/renal/cardiac function	· DFS, 72 months HR=1 (95% CI, 0.85 –1.17), *P=*0.974 • OS, 72 months • HR=0.98 (95% CI, 0.82 – 1.18), *P=*0.865	*p*<0.0001	· F sex HR=0.77 DFS, (95% CI, 0.58 – 1.01), *P=*0.019 HR=0.73 OS, (95% CI, 0.54 – 0.98), *P=*0.012 • Age<60 HR=0.88 DFS, (95% CI, 0.58 – 1.01), *P=*0.019 HR=0.86 OS, (95% CI, 0.65-1.13), *P=*0.301 • Stage IIIA HR=0.83 DFS, (95% CI, 0.61 – 1.12), *P=*0.549 HR=0.74 OS, (95% CI, 0.53–1.03), *P=*0.388 • N0 HR=0.74 DFS, (95% CI, 0.34 – 1.63), *P=*0.230 HR=0.61 OS, (95% CI, 0.26 – 1.45), *P=*0.845	Tx • Toxicity=83 (14.8) • Relapse/death= 11 (2) • Other=8 (1.4) C • Toxicity=30 (5.6) • Relapse/death= 20 (3.7) • Other=20 (3.7)	DFS: Exposure and control groups experience the same number of events across the 72-month period (recurrence and death). Adjuvant chemotherapy provided in the exposure group does not significantly affect DFS for stages II–III gastric cancer. OS: HR<1, but almost=1, meaning the exposure group experiences slightly lower event probability during a unit of time than the control group (tx=243 events [43.2%] versus C=240 events [44.6%]). No statistically significant benefit of adjuvant polychemotherapy (tx) versus single chemotherapy drug (C) on DFS and OS.
Noh *et al*. 2	(Tx) Capecitabine+Oxaliplatin 520 (50.2) (C) 515 (49.8)	· Age≥18 years • Ambulatory, Karnofsky performance≥70 • Stage II, IIIA, and IIIB • Gastrectomy with D2, within the past 6 weeks • ECOG<2 • No history of another cancer in the past 5 years except BCC skin and cured carcinoma *in situ* of uterine cervix. • No serious concomitant illness limiting life expectancy<5 years • No history of uncontrolled seizures, central nervous system disorders, or psychiatric disability. • No clinically significant cardiac disease within the past 12 months. • No physical integrity upper GI pathologies/malabsorption syndrome • No known peripheral neuropathy • No intercurrent uncontrolled infections • No moderate/severe renal impairment	· DFS, 75 months HR=0.58 (95% CI, 0.47–0.72), *P<*0.0001 • OS , 78 months HR=0.66 (95% CI, 0.52–0.85), *P=*0.0015	*p*<0.05	· Male sex HR=0.54 DFS, (95% CI, 0.42-0.70), *P=*0.25 HR=0.60 OS, (95% CI, 0.45 –0.81), *P=*0.1 • Age≥65 HR=0.51 DFS, (95% CI, 0.34-0.78), *P=*0.43 HR=0.70 OS, (95% CI, 0.44 – 1.12), *P=*0.86 • Stage II HR=0.55 DFS, (95% CI, 0.38 – 0.80), *P=*0.82 HR=0.54 OS, (95% CI, 0.34-0.87), *P=*0.55 • Stage IIIB HR=0.52 DFS, (95% CI, 0.33 – 0.82), *P=*0.82 HR=0.67 OS, (95% CI, 0.39–1.13), *P=*0.55 • T1, T2, T2a, and T2b HR=0.46 DFS, (95% CI, 0.33–0.74), *P=*0.05 HR=0.49 OS, (95% CI, 0.33–0.74), *P=*0.03 • N1 or N2 HR=0.57 DFS, (95% CI, 0.46–0.72), *P=*0.35 HR=0.67 OS, (95% CI, 0.51–0.87), *P=*0.71	Tx • Death=103 (20) • Adverse event including intercurrent illness=54 (10.4) • Recurrence=117 (23) C • Death=141 (27) • Adverse event including intercurrent illness=1 (0.19) • Recurrence=186 (36)	· DFS: HR<1, close to 0.50, meaning the exposure group provided with adjuvant chemotherapy experienced slightly more than half the event probability (death/recurrence) during unit of time than the control group over a 75-month period. • OS: HR<1, 0.66, meaning there is 34% less probability that an event will occur in the exposure group as compared to the control group. • There is a statistically significant benefit of adjuvant chemotherapy on both DFS and OS. As compared to control, the greatest effect of adjuvant chemotherapy is seen in DFS across 75 months.
Moon *et al*. 3	(Tx) 5-FU or 5’- DFUR or UFT, 113 (49.3) (C) 116 (50.7)	· Age<75 years • Stage IB, II, III with R0 resection within the past 6 weeks • No previous treatment for gastric cancer before the surgery • No organ function abnormalities (normal leucocyte, platelet hemoglobin, AST, ALT, blood urea nitrogen, and creatine concentration levels • Negative diagnostic peritoneal lavage	· RFS, 65 months HR=0.64 (95% CI, 0.33 – 1.20), *P=*0.163 • OS, 65 months HR=0.52 (95% CI, 0.25 – 1.04), *P=*0.063	*p*<0.05	· Stage II HR=0.36 OS, (95% CI, 0.12–0.94), *P=*0.036 • UFT chemotherapy HR=0.16 OS, (95% CI, 0.02 – 0.61), *P=*0.05	Tx • Cancer-related death=15 (13.3) • Other deaths=5 (4.4) • Recurrence=17 (15) C • Cancer-related death=20 (17.2) • Other deaths=6 (5.2) • Recurrence= 25 (21.6)	· RFS: HR<1, 0.64, meaning there is 36% less probability that an event will occur in the exposure group than in the control group over a 65-month period. • OS: HR<1, 0.52, meaning there is 48% (close to half) less probability that an event will occur in the exposure group than in the control group. • However, no statistically significant benefit of adjuvant chemotherapy on both RFS and OS (*p*>0.05) compared to surgery alone.
Kanda *et al*. 4	(Tx) S-1 79 (46.2) (C) 92 (53.8)	· Confirmed stomach adenocarcinoma • Stage II/III • R0 gastrectomy with D2 lymph- adenectomy • ECOG≤2 • No post-operative complications of poor oral intake within 6 weeks after surgery	· RFS, 60 months (Tx), %= 71 (C), %=53, *p*=0.030 • OS, 60 months (Tx), %=73, HR=2.42 (95% CI, 0.74 –10.8), *P=*0.165 (C), %=54, *P=*0.035	*p*<0.05	· Preoperative body mass index≥22 HR=0.74 OS, (95% CI, 0.24–2.23), *P=*0.588 • Tumor located in the lower third HR=0.89 OS, (95% CI, 0.24–2.74), *P=*0.846	Tx • Peritoneal recurrence=5 (5.7) C • Peritoneal recurrence=17 (18.6)	· RFS: Exposure group experienced 18% greater RFS, as compared to the control group, with statistical significance. • OS: Exposure group experienced 19% greater OS, as compared to the control. HR>1, 2.42, meaning there is approximately greater than twice the probability of an event (recurrence) occurring in the exposure group compared to the control. • Despite increased RFS and OS rates up to 19%, adjuvant chemotherapy provides no significant (p>0.05) reduction in event probability compared to surgery alone.
Lee *et al*. 5	(Tx) 5-FU or 5-FU with platinum or unknown 196 (31.1) (C) 434 (68.9)	· Stage IIA (T3N0M0, T2N1M0 or T1N2M0) • Curative gastrectomy with radical lymph node dissection within the past four to 6 weeks • >15 lymph nodes extracted • No 2° malignancies • No pre-operative chemotherapy/radiotherapy • ECOG≤2 • Normal blood values (neutrophils, platelets), no evidence of active infection • Normal renal and hepatic function • No systemic diseases contraindicating chemotherapy	· RFS, 84 months (Tx), %= 89.3 (C), %= 86.4, *p*=0.047 • OS, 84 months (Tx), %= 90.6 (C), %= 85.8, *P=*0.110	*p*<0.05	· T2N1 (96.8% RFS, *P<*0.001) (95.9% OS, *P=*0.003)	Tx • Recurrence=19 (9.7) C • Recurrence=55 (12.7) Total Deaths (Tx+C)=133 (21.2)	· RFS: Statistically, there is a significant difference showing exposure group experienced 2.9% greater RFS, as compared to the control group. • OS: Exposure group experienced 4.8% greater OS, as compared to the control, but there is no statistically significant difference in OS between exposure and control groups (*p*>0.05). • T2N1 responds most statistically significantly to adjuvant chemotherapy, with the greatest benefit on RFS over 84 months.
Cho *et al*. 6	(Tx) XELOX 114 (55.3) (C), TS-1 92 (44.7)	· Age≥18 years • Ambulatory • Stage III • R0 Gastrectomy with≥D2 lymph- adenectomy • No peritoneal, hepatic or distant metastases • Negative cytology for tumor cells in peritoneal fluid • No previous chemotherapy, radiotherapy, or immunotherapy for gastric cancer	· DFS, 51 months (Tx), %= 59.1 (C), %= 66.6, *p*=0.636 • OS, 51 months (Tx), %= 69.6 (C), %= 75.6, *P=*0.495	*p*<0.05	· Female sex HR=0.706 DFS, (95% CI 0.530 –1.966), *P=*0.606, HR=0.988 OS, (95% CI 0.356 – 2.745), *P=*0.434 • N1 HR=0.586 DFS, (95% CI 0.146 – 2.349), *P=*0.613, HR=0.989 OS, (95% CI 0.088–11.05), *P=*0.027	Tx • Adverse event=7 (6.1) • Recurrence=5 (4.4) C • Adverse event=4 (4.4) • Recurrence=11 (12)	· DFS: TS-1 (control) adjuvant chemotherapy achieves a 7.5% increase in DFS as compared to XELOX adjuvant chemotherapy (tx), over 51 months, but not statistically significant. • OS: Although statistically non-significant, the XELOX (tx) group achieves 6% greater OS over 51 months, as compared to TS-1 (C). • XELOX adjuvant chemotherapy provided to N1 patients has 0.586 DFS, meaning the probability of an event (adverse/recurrence) occurring is approximately 41.4% less as compared to TS-1. The OS for N1 patients receiving XELOX over control TS-1 is statistically significant (*p*<0.05).
Jin *et al*. 7	(Tx) S-1 or SOX or DOS or capecitabine+oxaliplatin 283 (17.7) (C) 1318 (82.3)	· Stage IA and IB (pT1N0M0, pT2N0M0) • Curative gastrectomy performed in past 8 years (between January 2011 and December 2017) • No co-occurring 1° malignancies or malignancies arising>6 months from gastric cancer diagnosis (metachronous disease) • No lymphoma, endocrine carcinomas, or stromal tumors • No anticipated serious complication response to chemotherapy	· CSS, 100 months HR=0.718 (95% CI, 0.508 – 1.014), *P=*0.060 • OS, 100 months HR=0.940 (95% CI, 0.710 – 1.245), *P=*0.688	*p*<0.05	· Body (lesser curvature) HR=0.56 CSS, (95% CI 0.220 – 1.422), *P=*0.223, HR=0.577 OS, (95% CI 0.298 – 1.116), *P=*0.102 • Diffuse -type HR=0.88 CSS, (95% CI 0.563 – 1.376), *P=*0.576, HR=0.644 OS, (95% CI 0.458 –0.906), *P=*0.011 • T2N0M0 with examined lymph nodes≤15, tumor size>3cm, Non-SRCC (CSS, *P=*0.034)	(None reported) Tx+C • No follow-up=128 (18)	· CSS: HR<1, 0.718, meaning there is approximately 28% less probability that an event will occur in the exposure group as compared to the control group, over 100 months. However, *P>*0.05 (*p*=0.060), so statistically not significant. • 4.8% greater OS over 51 months, as compared to TS-1 (C). • OS: HR<1, 0.940, but close to 1. Meaning there is only 6% less probability an event will occur in the exposure group compared to the control group over 100 months. *P>*0.05 (*p*=0.060), so statistically not significant. • Statistically significant responses to adjuvant chemotherapy are seen in CSS of T2N0M0 (non-SRCC), with examined lymph nodes≤15 and tumor size>3 cm *(p=*0.034). Statistically significant positive response to adjuvant chemotherapy is also seen in OS of patients with diffuse-type gastric cancer (0.644 OS, 95% CI 0.458–0.906, *P=*0.011).
Huang *et al*. 8	(Tx) 287 (18) (C) 1,306 (82)	· Stage IIA (T1N2M0, T3N0M0) • Radical gastrectomy performed • Only 1° site of the tumor in the stomach • Patients entered into the SEER database (1988 to 2012), FMUUH database (2008 to 2014), and IMIGASTRIC database (2000 to 2014) with complete demographic, staging, and survival information • No distant metastasis • No radiotherapy provided	· OS, 400 months *P=*0.0001, *P=*0.012, *P=*0.042	*p*<0.05	· T1N2M0 HR=0.56 OS, (95% CI 0.359 – 0.871), *P=*0.010 • LN≤15 dissected HR=0.602 OS, (95% CI 0.414 –0.875), *P=*0.008 • Tumor size≥20 mm HR=0.527 OS, (95% CI 0.336 – 1.234), *P=*0.009	Tx • Bleeding loss≤90 to>90 mL=159 (55.4) C • Bleeding loss≤90 to>90mL=127 (9.7)	· OS: Throughout the study (approximately 400 months), OS of patients across all databases was statistically significantly better in the exposure group (*p*<0.05). • Adjuvant chemotherapy has a statistically significant benefit on the overall survival of Stage IIA patients. T1N2M0, ≤15 LN dissected and tumor size≥20 mm afford lowest HR (<1), meaning the lowest probability of event likelihood, compared to control.
Lee *et al*. 9	(Tx) 768 (78.1) (C) CAPOX 215 (21.9)	· Age ≥20 years • Stage II/III • Radical Gastrectomy • Received adjuvant chemotherapy within 8 weeks of surgery • No pre-operative chemotherapy, radiotherapy, or immunotherapy • No distant metastasis • No co-occurring 1° malignancies or malignancies arising >6 months from gastric cancer diagnosis (metachronous disease) • Negative cytology for tumor cells in peritoneal fluid	· DFS, 60 months Tx, (%) 66.1 (95% CI=64.3–67.9), *P=*0.961 C, (%) 66.5 (95% CI=62.5–70.5), *P=*0.961 HR=1.008 (95% CI, 0.728 – 1.395), *P=*0.963	*p*<0.05	· Age≥60 (*p*=0.014) • T3 (*p*=0.045, *p*=<0.001) • T4 (*p*=0.007) • Tumor size>5 cm (*p*<0.001) • Vascular invasion (*p*=0.001) • Lymphatic invasion (*p*=0.012)	Tx • Recurrence=141 (32.9) C • Recurrence=49 (31.6)	· DFS: Although statistically insignificant, over a 60-month duration, tx with chemotherapeutic regimen S-1 achieves 0.4% less DFS than C, CAPOX chemotherapeutic regimen. • HR>1, meaning there is no significant difference in the probability of events occurring, that is, recurrence (specifically peritoneal, hematogenous, lymph node, and locoregional) across both groups. • Significant responses to adjuvant chemotherapy in both groups include age≥60, T3 and T4, tumor size>5 cm, and vascular/lymphatic invasion. Overall, there is no distinction between which regimen (tx, S-1) or (C, CAPOX) affords statistically significant benefit as adjuvants for Stage II/III.
Chen *et al*. 10	(Tx) SOX, XELOX, FOLFOX in 7–8/10–12 cycles (Group B), 4–6/6–9 cycles (Group C),≤three or five cycles (Group D), 342 (79.9) (C), N (%) = 86 (20.1)	· Age 18 – 79 years • Radical gastrectomy with D2 lymphadenectomy Ambulatory • Stage II–III • Receipt of adjuvant chemotherapy within 3 months after surgery • No history of other malignancy • No prior adjuvant radiation or neoadjuvant chemotherapy • No prior receipt of adjuvant chemotherapy (mono/dual/triplet)	· DFS, 60 months Tx, (%) Group B=68 (95% CI=61–75) Group C=65.4 (95% CI=56.3–74.6) Group D=50 (95% CI=37–63), C, (%) 50 (95% CI=39.2–60.8) • OS, 60 months Tx, (%) Group B=73.7 (95% CI=67.1–80.3) Group C=72 (95% CI=63.3–80.6) Group D=53.3 (95% CI=40.3–66.3) C, (%)=52.3 (95% CI=41.6–63.1)	*p*<0.008	· Group B, compared to Group D and (C), DFS *P=*0.003 HR=0.459 OS, (95% CI 0.301 – 0.699), *P<*0.001 • Group C compared to Group D and (C), OS *P=*0.004 and *P=*0.005 HR=0.504 OS, (95% CI 0.314 –0.807), *P=*0.004	Tx • Nausea/vomiting=52 (16.2) • Neutropenia=60 (18.7) • Thrombocytopenia=21 (6.5)	DFS: ⅔ of exposure groups (Groups B and C) achieve 15.4–18% greater DFS rates than control, over 60 months. OS: All exposure groups (Groups B, C, and D) achieve 1–21.4% greater OS rates than control, over 60 months. Group B (7–8/10–12 cycles of tx) and C (4–6/6–9 cycles of tx) have HR<1, close to 0.5, meaning approximately half of the probability of adverse events, as compared to control. The DFS response for Group B and the OS response of Group C, to adjuvant chemotherapy, are statistically significant *(p<*0.008).
Kang *et al*. 11	(Tx), UFT, TS-1, 5-FU, FL, FP or XELOX 612 (56.5) (C) 471 (43.5)	· Stage II (pT3N0M0) • Curative Gastrectomy with radical lymph- adenectomy • No neoadjuvant chemotherapy • No remnant gastric cancer • No co-occurring 1° malignancies or malignancies arising>6 months from gastric cancer diagnosis (metachronous disease) • Tumor median size 4.5 cm and histologically classified	· DFS, 59 months Tx, (%) 89.9 C, (%) 89.2 *P>*0.05 • Tumor recurrence HR=0.925 (95% CI=0.625–1.368), *P=*0.695	*p*<0.05	· (Tx) FP HR=0.740 (95% CI=0.349–1.569), *P=*0.432 • (Tx) TS-1 HR=0.623 (95% CI=0.336–1.154), *P=*0.133	Tx • Recurrence=99 (16.2) C • Recurrence=49 (31.6)	· DFS: Although statistically insignificant, over a 59-month duration, tx group experiences 0.7% higher DFS than the control of no adjuvant chemotherapy. • For tumor recurrence, HR<1 but almost 1, with no statistical significance, meaning there is no difference in the probability of events (i.e., tumor recurrence) among the tx and C groups. • Individual chemotherapeutic tx (FP and TS-1) afford HR <1 meaning less probability of events (i.e., recurrence occurring); however, this is not statistically significant. • Thus, there is no statistically significant benefit of adjuvant chemotherapy tx over control for pT3N0M0.
Serrano *et al*. 12	(Tx), Capecitabine+oxaliplatin 100 (57.8) (C) 73 (42.2)	· Age≥18 years • Stage II–III with radical gastrectomy and D2 lymph-adenectomy performed between 2014 and 2016. • Normal blood values (normal neutrophil/ lymphocyte ratio, albumin≥3.5 g/dL, albumin/globulin ratio>1.5)	· DFS, 46 months Tx, (%) 69 C, (%) 52.6 *P=*0.034 HR=0.28 (95% CI=0.17–0.47), *P<*0.001 • OS, 48 months Tx, (%) 73 C, (%) 57.5 *P=*0.027 HR=0.27 (95% CI=0.16–0.46), *P<*0.001	*p*<0.05	· Age≥65 DFS HR=0.43 (95% CI=0.19–0.97), *P=*0.04 • pN2 DFS HR=0.13 (95% CI=0.0349–0.452), *P<*0.01 • Stage IIIA DFS HR=0.14 (95% CI=0.0505–0.375), *P<*0.01	Tx • Death=31 (31) • Adverse events (neutropenia, thrombocytopenia, anemia nausea, diarrhea, peripheral neuropathy, asthenia, hand-foot syndrome)=85 (85) C • Death=34 (46.6)	· DFS: Tx offers 16.4% greater DFS over 46 months, with statistical significance (*p*<0.05). HR=0.28, meaning the probability of an event is 72% less in tx compared to control, with statistical significance. • OS: Tx offers 15.5% greater OS over 48mo, with statistical significance (*p*<0.001). HR=0.27, meaning the probability of an event is 73% less in tx compared to control, with statistical significance. • Statistically significant factors that favor tx, in terms of DFS, include age≥65, pN2, and stage IIIA. Thus there is statistically significant benefit on DFS and OS of adjuvant chemotherapy across stages II–III.
Mei *et al*. 13	(t) S-1+XELOX or SOX 196 (63.8) (C) 111 (36.2)	· Age<80 years • Radical gastrectomy with D2 lymph- adenectomy between 2012–2016 • No previous gastric surgery • Stage T2N0M0 • Only R0 surgical margins (no R1 or R2) • ≥15 harvested lymph nodes • No neoadjuvant chemotherapy • No other 1° malignancies	· DSS, 5y Tx, (%) 83 C, (%) 96.8 *P<*0.001 • OS, 5y Tx, (%) 92.9 C, (%) 79.3 *P<*0.001	*p*<0.05	· Mono-therapy tx (5-year DSS (%)=95.2, *P=*0.083) • Dual drug tx (5-year DSS (%)=100, *P=*0.083)	Tx • Death due to recurrence=6 (0.25) C • Death due to recurrence=18 (0.75)	· DSS: Tx offers 13.8% greater DSS over 5y with statistical significance (*p*<0.001). • OS: Tx offers 13.6% greater OS over 5 years with statistical significance (*p*<0.05). • Factors favoring tx include a 5-year DSS rate of 95.2% for monotherapy tx and 100% for dual drug tx, although both these results are *P>*0.05, therefore not statistically significant. • For patients with T2N0M0, a statistically significant benefit of adjuvant chemotherapy can be seen in DSS and OS over 5 years.
Wada *et al*. 14	(Tx), <9 months of S-1 26 (33.8) (C), ≥9 months of S-1 N (%) = 51 (66.2)	· Histologically proven gastric adenocarcinoma • Stage II/III • Gastrectomy, R0, with ≥D1 – D2 lymph- adenectomy, between 2003–2008 (past 10 years) • Adjuvant S-1 chemotherapy after surgery • No previous treatment for cancer except surgery for 1° tumor	· RFS, 12 months OR=0.567 (95% CI, 0.242 – 1.329), *P=*0.192 • Recurrences Tx, N(%)=10 (38.5), *P=*0.195 C, N(%)=13 (25.5), *P=*0.195	*p*<0.05	· N0-2 (p=0.036) • Tumor size<5 cm (*p*=0.020)	Tx • Anorexia=10 (38.5) • Fatigue=3 (11.5) • Diarrhea=2 (7.7) • Neutropenia=2 (7.7) • Rash=2 (7.7) • Recurrence=1 (3.8) C • Anorexia=1 (2) • Neutropenia=1 (2) • Rash=1 (2) • Alopecia=1 (2)	· RFS: Although statistically insignificant, over a 12-month duration, tx with<9 months of S-1 achieves odds 0.567 times (approximately half) the estimated odds of recurrence than >9 months of S-1. • Recurrence rates are 13% less in tx (<9 months of S-1), compared to C (>9 months of S-1), although no significant difference between groups (*p*>0.05). • Significant responses to tx (<9 months) of adjuvant S-1 chemotherapy include N0-2 (*p*=0.036) and tumor size<5 cm (*p*=0.020).
Aoyama *et al*. 15	(Tx), T1N2M0 and T1N3M0 28 (53.8) (C), T3N0M0 24 (46.2)	· Histologically proven gastric adenocarcinoma • Stage II (T1N2M0, T1N3M0, T3N0M0) • Total/distal gastrectomy performed with D1+to D2 lymphadenectomy between 2000 and 2010 • No other adjuvant chemotherapy received after surgery	· RFS, (%) 3 years T1N2M0=93.3 T1N3M0=91.7 • T3N0M0=100 6 years T1N2M0=80 T1N3M0=76.4 T3N0M0=100	*p*<0.05	· Tumor diameter≥30 to≤50 mm (3 year RFS 100%, 5 year RFS 100%, *P=*0.0449) • Positive lymphatic invasion OR=0.341, (95% CI 0.035–13.302), *P=*0.353	(Not specified)	· RFS: Across both groups, 3-year and 5-year RFS remained high (80–100%). T3N0M0 affords 100% RFS over 3 years and 5 years. Tumor diameter ≥30 to≤50 mm affords 100% RFS with statistical significance, across both groups. Although statistically not significant, tumors with positive lymphatic invasion, have OR of 0.341 meaning the odds of recurrence in T1N2M0/T1N3M0 are 0.341 times compared to T3N0M0. • Thus, according to statistical significance, larger diameter tumors with T3N0M0 may achieve greater RFS with adjuvant chemotherapy.
Kano *et al*. 16	(Tx), S-1 390 (43) (C), S-1 ACTS-GC Trial 517 (57)	· Age≤81 • Stage II/III • R0 Gastrectomy performed with>D2 lymphadenectomy between 2008 and 2012 • No neoadjuvant chemotherapy received • No synchronous cancer • No remnant gastric cancer after surgery • No histological special types • Eligible to receive adjuvant chemotherapy (good performance statement, physician’s decision, patient’s wish, no early recurrence, etc.) • Did not receive other adjuvant chemotherapy regimens than S-1 monotherapy for example, capecitabine+oxaliplatin and S-1+cisplatin.	· RFS, 5 year tx, (%) II=85.9,86.4 IIIA=68.1, 73.9 IIIB=51.6, 46.3 C, (%) II=79.2, 77.9 IIIA=61.4, 64.3 IIIB=37.6, 35.9 • OS, 5 year tx, (%) II=86.6,87.6 IIIA=73.6, 79.1 IIIB=57, 50 C, (%) II=84.2,83.4 IIIA=67.1, 69.1 IIIB=50.2, 44. 8	(Not specified)	(N/A)	Tx • Adverse effects=70 (17.9) • Recurrence=115 (29.5) C • Adverse effects=72 (13.9) • Recurrence=162 (30.6)	· RFS: Across all individual stages (II, IIIA, IIIB), RFS rates over 5 years are higher in the tx S-1 group, as compared to the ACTS-GC trial control. The highest RFS rate in the tx group is noted in stage II (8.5% higher than control). The highest difference in RFS rates is noted in stage IIIB (tx group achieves 14% greater RFS than the control). OS: Across all individual stages (II, IIIA, IIIB), OS rates over 5 years are higher in the tx S-1 group, as compared to the ACTS-GC trial control. The highest RFS rate in tx group is noted in stage II (4.2% higher than control). The highest difference in RFS rates is noted in stage IIIA (tx group achieves 10% greater OS than the control). • Based on differences in RFS and OS rates, stages IIIA and IIIB may respond more significantly to tx, affording higher survival. • Although there are no statistical *P* values for reference to comment on the significance of this association.
Takahashi *et al*. 17	(Tx), recurrence after S-1 122 (30.4) (C), S-1 396 (98.8)	· Stage II/III (excluding pT1N0M0 and pT3N0M0) • Curative gastrectomy with≥D2 lymphadenectomy (distal or total) between 2008 and 2012. • No neoadjuvant chemotherapy • No remnant gastric cancer • Eligible for S-1 (physician discretion, patient consent, contraindicated other treatments for other diseases	· RFS, (%) 1 year=67.2 3 years=23 5 years=5.7 Median RFS, months (range, IQR)=19.5 (2–78, 10–33) *P=*0.719 • OS, (%) Median OS, months (range, IQR)=10 (0–100, 5–19.5) *p*=0.311	*p*<0.05	· Local recurrence (100%) and lymphatic recurrence (92%) in tx for recurrence patients plateaus 3 years after gastrectomy • Stage IIIA (median RFS, months=24, *P=*0.719)	Tx+C • Recurrence during chemo- therapy=22 (5.5) • Recurrence after 1 year tx=14 (3.5) • GI toxicity=13 (3.2) • Hematological events=3 (0.7) • Eczema=1 (0.2) • Ophthalmopathy=1 (0.2)	· RFS: The highest RFS rate is seen 1 year post-adjuvant chemotherapy in the exposure group (patients who have experienced recurrence post-curative gastrectomy), compared to three and 5 years. • OS: Median OS across all stages is 9.5 months less than median RFS, meaning patients given adjuvant chemotherapy after recurrence from surgery will experience greater RFS. Median RFS is notably highest in Stage IIIA; however, *P>*0.05 across all values is statistically insignificant. • Recurrence patterns plateau in the tx group when chemotherapy is given up to 3 years. • Paper site’s times to recurrence, expressed as higher RFS/OS rates, were better compared to the literature; however, raw data was not provided to make a statistical comparison.

Abbreviations: ACTS-GC: Adjuvant chemotherapy trial of TS-1 for gastric cancer; ALT: Alanine aminotransferase; AST: Aspartate aminotransferase; BCC: Basal cell carcinoma; CI: Confidence interval; CSS: Cause-specific survival; DFS: Disease-free survival; DSS: Disease-specific survival; ECOG: Eastern Cooperative Oncology Group, FMUUH: Fujian Medical University Union Hospital; GI: Gastrointestinal; HR: Hazard ratio; IMIGASTRIC: International Study Group on Minimally Invasive Surgery for Gastric Cancer; IQR: Interquartile range; OR: Odds ratio; OS: Overall survival; RFS: Recurrence-free survival; SEER: Surveillance, Epidemiology, End results.

**References**

1. Bajetta E, Floriani I, Bartolomeo DM, *et al*. Randomized trial on adjuvant treatment with FOLFIRI followed by docetaxel and cisplatin versus 5-fluorouracil and folinic acid for radically resected gastric cancer. *Ann Oncol*. 2014;25(7):1373-1378.
  doi: 10.1093/annonc/mdu146
2. Noh HS, Park RS, Yang KH, *et al*. Adjuvant capecitabine plus oxaliplatin for gastric cancer after D2 gastrectomy (CLASSIC): 5-year follow-up of an open-label, randomised phase 3 trial. *Lancet Oncol*. 2024;15(12):1389-1396.
  doi: 10.1016/S1470-2045(14)70473-5
3. Moon HJ, Fujiwara Y, Hirao M, *et al*. Randomized controlled trial of adjuvant chemotherapy with fluoropyrimidines versus surgery-alone for gastric cancer. *Anticancer Res*. 2017;37(6):3061-3067.
4. Kanda M, Murotani K, Kobayashi D, *et al*. Postoperative adjuvant chemotherapy with S-1 alters recurrence patterns and prognostic factors among patients with stage II/III gastric cancer: A propensity score matching analysis. *Surgery*. 2015;158(6):1573-1580.
  doi: 10.1016/j.surg.2015.05.017
5. Lee KG, Lee HJ, Oh SY, *et al*. Is there any role of adjuvant chemotherapy for T3N0M0 or T1N2M0 gastric cancer patients in stage II in the 7^th^ TNM but stage I in the 6^th^ TNM system? *Ann Surg Oncol*. 2016;23:1234-1243.
  doi: 10.1245/s10434-015-4980-7
6. Cho HJ, Lim YJ, Cho YJ. Comparison of capecitabine and oxaliplatin with S-1 as adjuvant chemotherapy in stage III gastric cancer after D2 gastrectomy. *PLoS One*. 2017;12(10):e0186362.
  doi: 10.1371/journal.pone.0186362
7. Jin P, Ji X, Ma S, *et al*. Adjuvant chemotherapy indications for stage I gastric cancer patients with negative lymph node. *Clin Res Hepatol Gastroenterol*. 2021;45(6):101634.
  doi: 10.1016/j.clinre.2021.101634
8. Huang NZ, Desiderio J, Chen YQ, *et al*. Indications for adjuvant chemotherapy in patients with AJCC stage IIa T3N0M0 and T1N2M0 gastric cancer-an East and West multicenter study. *BMC Gastroenterol*. 2019;19:205.
  doi: 10.1186/s12876-019-1096-8
9. Lee MC, Yoo WM, Son GY, *et al*. Long-term efficacy of S-1 monotherapy or capecitabine plus oxaliplatin as adjuvant chemotherapy for patients with stage II or III gastric cancer after curative gastrectomy: A propensity score-matched multicenter cohort study. *J Gastric Cancer*. 2020;20(2):152-164.
  doi: 10.5230/jgc.2020.20.e13
10. Chen L, Zhang C, Yao Z, *et al*. Adjuvant chemotherapy is an additional option for locally advanced gastric cancer after radical gastrectomy with D2 lymphadenectomy: A retrospective control study. *BMC Cancer*. 2021;21(1):974.
  doi: 10.1186/s12885-021-08717-4
11. Kang M, Youn GH, An YJ, *et al*. Adjuvant chemotherapy vs. surgery alone for pT3N0M0 gastric cancer. *Ann Surg Oncol*. 2021;28:1437-1444.
  doi: 10.1245/s10434-020-09063-8
12. Serrano M, Araujo MJ, Pacheco C, *et al*. Adjuvant chemotherapy after curative D2 gastrectomy in Latin American patients with gastric cancer. *Ecancermedicalscience*. 2022;16:1387.
  doi: 10.3332/ecancer.2022.1387
13. Mei Y, Feng X, Feng T, *et al*. Adjuvant chemotherapy in pT2N0M0 gastric cancer: Findings from a retrospective study. *Front Pharmcol*. 2022;13:845261.
  doi: 10.3389/fphar.2022.845261
14. Wada T, Kunisaki C, Hasegawa S, *et al*. Factors predictive of recurrence after surgery for gastric cancer followed by adjuvant S-1 chemotherapy. *Anticancer Res*. 2013;33:1747-1752.
15. Aoyama T, Yoshikawa T, Fujikawa H, *et al*. Exploratory analysis to find unfavourable subset of stage II gastric cancer for which surgery alone is the standard treatment; Another target for adjuvant chemotherapy. *Int Surg*. 2014;99(6):835-841.
  doi: 10.9738/INTSURG-D-13-00176.1
16. .Kano Y, Ohashi M, Hiki N, *et al*. Favourable long-term outcomes of one-year adjuvant S-1 monotherapy for pathological stage II or III gastric cancer treated at a high-volume center. *Gastric Cancer*. 2018;21:1024-1030.
  doi: 10.1007/s10120-018-0827-9
17. Takahashi R, Ohashi M, Kano Y, *et al*. Timing and site-specific trends of recurrence in patients with pathological stage II or III gastric cancer after curative gastrectomy followed by adjuvant S-1 monotherapy. *Gastric Cancer*. 2019;22:1256-1262.
  doi: 10.1007/s10120-019-00953-9
